# Oral Potentially Malignant Disorders among Patients Attending the Department of Oral Medicine and Radiology of a Tertiary Care Dental Hospital: A Descriptive Cross-sectional Study

**DOI:** 10.31729/jnma.7494

**Published:** 2022-05-31

**Authors:** Deepa Gurung, Ujjwal Joshi, Bikash Chaudhary, Preeti Singh

**Affiliations:** 1 Department of Oral Medicine and Radiology, Kathmandu Medical College, Duwakot, Bhaktapur, Nepal; 2 Department of Oral and Maxillofacial Surgery, Kathmandu Medical College, Duwakot, Bhaktapur, Nepal; 3 Department of Oral Pathology, Kathmandu Medical College, Duwakot, Bhaktapur, Nepal

**Keywords:** *Nepal*, *oral cancer*, *prevalence*, *tobacco*

## Abstract

**Introduction:**

Despite oral cancer being a common cancer in Nepal, little is known about oral potentially malignant disorders which have a high likelihood of malignant transformation. This study aims to find out the prevalence of ora! potentially malignant disorders among patients attending the Department of Oral Medicine and Radiology of a tertiary care dental hospital.

**Methods:**

A descriptive cross-sectional study was carried out among 611 patients from 14^th^ January, 2021 to 15^th^ July, 2021 at the Department of Oral Medicine and Radiology of a tertiary care centre after receiving ethical clearance from the Institutional Review Committee (Reference number: 2306202002). Convenience sampling was done. Patients aged 18 years and above were enrolled into the study. Follow-up cases and patients requiring emergency treatment were, however, excluded. All demographic data, cancer and dietary history and clinical examinations were recorded. Descriptive analysis was done using Statistical Package for the Social Sciences version 22.0. Point estimate was done at a 95% Confidence Interval for frequency and percentages for binary data.

**Results:**

Among 611 total participants, the prevalence of oral potentially malignant disorders was found to be 26 (4.25%) (2.65-5.85 at a 95% Confidence Interval), where males were 19 (73.07%), females were 7 (26.92%), and 23 (88.46%) of them had history of consuming various forms of tobacco, areca nut, and alcohol.

**Conclusions:**

The prevalence of oral potentially malignant disorders in this study was found to be lower than previous studies conducted in similar settings.

## INTRODUCTION

Globally, cancers of lip and oral cavity are on the rise with 377,713 new cases and 177,757 deaths in 2020.^1^ Their incidence is particularly high in low- and middle-income countries in Asia, especially among males, due to high consumption of tobacco products and alcohol.^1,2^ In addition, most oral cancers are diagnosed late with five years of survival rate in 60% cases.^3^ Oral cancers are often preceded by Oral Potentially Malignant Disorders (OPMD), therefore early detection and treatment of OPMDs can lead to a reduction in progression and incidence of oral cancers.^4^

Although cancer of the lip and oral cavity is a common cancer in Nepal, little is known about the prevalence of OPMD in Nepal, with limited studies on its risk factors.^5^

The objective of this study is to find out the prevalence of OPMD among patients attending the Department of Oral Medicine and Radiology of a tertiary care dental hospital.

## METHODS

A descriptive cross-sectional study was carried out among adult patients who visited the Department of Oral Medicine and Radiology of Kathmandu Medical College (KMC), Duwakot from 14^th^ January, 2021 to 15^th^ July, 2021. Ethical approval was obtained from the Institutional Review Committee of KMC (IRC No: 2306202002) before conducting this study. Patients of both sexes, aged 18 years and above who gave their consent were included in our study, whereas patients who came for follow-ups and/or patients who came for emergency treatment were excluded. Convenience sampling was done and sample size was calculated using the following formula,

n = (Z^2^ × p × q) / e^2^

  = (1.96^2^ × 0.1463 × 0.8537) / 0.03^2^

  = 534

Where,

n = minimum required sample size
Z = 1.96 at 95% Confidence Interval (CI)
p = prevalence of oral potentially malignant disorders, 14.63%^5^
q = 1-p
e = margin of error, 3%

The minimum required sample size was 534. Ten percent was added to address the nonresponse rate, after which a minimum required sample size of 593 was calculated. However, a sample of 611 was taken. Data collection was done through a set of questionnaires that recorded sociodemographic details, history of cancer/oral cancer as well as family history of cancer, and participant's deleterious habit history such as the habit of tobacco smoking, chewing smokeless tobacco, gutkha, areca nut, alcohol consumption, etc. A thorough clinical examination of hard and soft tissues of the oral cavity was then carried out by a single oral medicine specialist. Any OPMD such as leukoplakia, erythroplakia, oral submucous fibrosis, oral lichen planus, actinic cheilitis was diagnosed clinically.

All recorded data were extracted to a Microsoft Excel Sheet and then further analysed using Statistical Package for Social Sciences (SPSS) version 22.0. Point estimate was done at a 95% Confidence Interval for frequency and percentages for binary data.

## RESULTS

Among 611 total participants, the prevalence of oral potentially malignant disorders was found to be 26 (4.25%) (2.65-5.85 at a 95% Confidence Interval), where males were 19 (73.07%), females were 7 (26.92%), and 23 (88.46%) of them had history of consuming various forms of tobacco, areca nut, and alcohol.

Leukoplakia 10 (1.64%), oral submucous fibrosis 10 (1.64%), and oral lichen planus 6 (0.98%) were the only three lesions diagnosed as OPMD (Table 1).

**Table 1:** Distribution of various types of oral potentially malignant disorders (n = 26).

Types of OPMD	n (%)
Leukoplakia	10 (1.64)
Oral submucous fibrosis	10 (1.64)
Oral lichen planus	6 (0.98)
Total	26 (4.25)

Among 26 OPMD participants, 23 (88.46%) of them were married, and 24 (92.30%) were Hindu in religion with their mean age around 46 years (Table 2).

**Table 2:** Demographic details in OPMD individuals (n = 26).

Characteristics	n (%)
**Gender**	
Males	19 (73.07)
Females	7 (26.92)
**Marital status**	
Married	23 (88.46)
Unmarried/Single	3 (11.54)
**Religion**	
Hindu	24 (92.30)
Buddhism	2 (7.69)
**Education status**	
Postgraduate or above degree completed	1 (3.85)
Bachelor's degree completed	16 (61.54)
High school completed	3 (11.54)
Secondary school completed	6 (23.07)

On deleterious habit history, 23 (88.46%) OPMD participants gave positive history of consuming various forms of tobacco, areca nut and/or alcohol on regular or occasional basis (Table 3).

**Table 3:** Deleterious habit history (n = 26).

Distribution of various form of deleterious habits	Yes n (%)	Occasionally n (%)
Tobacco smoking	13 (50)	1 (3.85)
Chewing smokeless tobacco	3 (11.54)	1 (3.85)
Gutkha	10 (38.46)	2 (7.69)
Areca nut	2 (7.69)	-
Alcohol	-	7 (26.92)
No deleterious habit	3 (11.5)	

## DISCUSSION

In this hospital-based study, the prevalence of OPMD among adult patients was found to be 4.25%, which was considerably more among males and those with deleterious tobacco/alcohol habits. The study findings are similar to the global prevalence of OPMD of 4.47% as reported by a meta-analysis conducted in 2018.^6^ However, the same study also identified Asian countries to have the highest prevalence of OPMD at 10.54% (95% CI = 4.60-18.55) as compared to other countries in the world.^6^ Similarly, a study reported a high OPMD prevalence of 14.6% in the eastern developmental region of Nepal.^5^ These differences could be attributed to differential tobacco consumption between the study participants, availability of local products, and lack of oral health awareness in various countries or various regions of the same country.

OPMD were previously categorised as precancerous or premalignant lesions and conditions as those lesions and conditions were believed to be the early signs of oral cancer.^7^ However, these lesions do not necessarily progress into oral cancer and rather indicate only the higher chances of oral cancer occurrence. They were therefore replaced with the term 'OPMD' in 2005.^7^ OPMD includes various red and white lesions of oral mucosa such as leukoplakia, erythroplakia, Oral Submucous Fibrosis (OSMF), Oral Lichen Planus (OLP) and Actinic Cheilitis (AC).^6^ OPMD is also called as 'Potentially Premalignant Oral Epithelial Lesions (PPOEL)' by some recent authors.^8,9^

Several risk factors are associated with the occurrence of OPMD, such as consuming different forms of tobacco, areca nut, drinking alcohol, human papillomavirus infection, chronic exposure to ultraviolet radiation, and genetic abnormalities.^6,10^ Among those, the most common risk factors include chewing areca nut with or without tobacco.^5^ Previous studies indicate that 50% of oral cancers in India are caused by areca nut/tobacco chewing and smoking habits and 30% of oral cancers are attributed to areca nut/tobacco chewing habits alone.^11^

Although, Tobacco Control and Regulation Act in 2011 enforced a law in Nepal which included the prohibition of consuming tobacco in public areas, prohibition of tobacco sale to children under 18 years, and mandatory pictorial and textual warnings on tobacco packets' cover,^12^ our study still showed a high proportion of participants consuming various forms of tobacco. A prior study had shown a 18.6% prevalence of smokeless tobacco in Nepal, where the participants aged as young as 15 years were reported to be consuming smokeless tobacco.^12^ These findings necessitate a strict implementation of tobacco prohibitory rules, both in rural and urban areas, as well as oral health awareness education to be introduced at the school level curriculum.

Although most OPMD can be diagnosed clinically, histopathological examination remains the gold standard for its diagnosis.^6^ Oral squamous cell carcinoma is the most common oral cancer diagnosed histopathologically. A study in Nepal showed more than two-thirds of oral squamous cell carcinoma patients were in stages III and IV of the disease,^3^ which again suggests the lack of oral health awareness as well as the late diagnosis of the disease. This may be reduced by organising various oral health awareness programs, broadcasting awareness videos and messages through various media, and encouraging routine oral health check-ups.^13^ Likewise, proper examination of all oral mucosa should be focused by all general dental health practitioners during routine dental check-ups, ensuring timely referral to oral medicine specialists for any doubtful lesions.

There are a few limitations to our study. Firstly, this is a single hospital-based study and may not be representative of all adult populations of Nepal. While prevalence of disease is best understood with population-based studies, large population-based surveys are limited in a developing country like Nepal. Secondly, all diagnoses in this study were made on the basis of patient history and clinical examination only. This could have allowed for a slight variation of the prevalence as the final diagnosis may differ in a few cases after histopathological investigation.

## CONCLUSIONS

The prevalence of OPMD in this hospital-based study was found to be lower than previous studies conducted in similar settings. Nevertheless, the study findings indicate a need for reducing tobacco consumption and increasing oral health awareness programs in Nepal. Large population-based studies with histopathological confirmation are required to have a better understanding of the true prevalence of OPMD in Nepal.
